# The first record of biological activities and chemical constituents of different extracts and semi-purified fractions of *Bidens aurea* (Aiton) Sherff (Asteraceae)

**DOI:** 10.1186/s12906-025-04924-9

**Published:** 2025-06-02

**Authors:** Shaimaa S. Shoman, Randa S. Hasan, Rim Hamdy, Emad A. Shalaby

**Affiliations:** 1 Faculty of Agriculture, Department of Biochemistry, Cairo University, Giza, 12613 Egypt; 2 Regional Center for Food and Feed (RCFF), Agricultural Research Center (ARC), P. Box 588, Orman, Giza, Egypt; 3 Department of Botany and Microbiology, Faculty of Science, Cairo University, Giza; Biological Sciences Department, Faculty of Science, Galala University, Suez, Egypt

**Keywords:** *Bidens aurea*, Phytoconstituents, Natural antioxidant, Antimicrobial potential, GC–MS/MS

## Abstract

*Bidens aurea* is a flowering plant known for its yellow or white flowers rich with various bioactive chemical compounds that possess or have proven high medicinal values, This research aimed to investigate the in vitro antioxidant and antimicrobial abilities of individual polar extracts and semi-purified fractions of *Bidens aurea*. In this study, three different extracts (cold water, hot water, and ethanol) and 6 isolated fractions of ethanolic extract (as the most potent sample) were assessed for their antiradical and antioxidant properties in comparison with ascorbic acid a natural standard with 2,2-diphenyl- 1-picrylhydrazyl (DPPH^•^) and 2,2-azinobis (3-ethylbenzothiazoline- 6-sulfonic acid) (ABTS) radical scavenging assays, in addition to, GC–MS/MS identification of fractions and biochemical compounds. The findings revealed that, both methods were correlated in which the ethanolic extract showed the highest antioxidant effect with 61.71 ± 0.22 and 87.29 ± 0.56% in comparison with ascorbic acid as the natural standard (95.12 ± 0.29 and 99.32 ± 0.08%) at 1000 µg/ml against DPPH and ABTS, respectively. The fraction no. 6 (ethanol 100%) recorded the highest antioxidant activity by 90.40 ± 0.13 and 94.34 ± 0.18% at 1000 µg/ml respectively followed by fraction no. 5 (ethyl acetate: ethanol 20:80) by 82.61 ± 0.19 and 87.00 ± 0.48% at 1000 µg/ml, respectively. The antimicrobial effect revealed that the ethanolic extract of *Bidens aurea* was effective against all tested microorganisms with growth inhibition zones (IZ) diameters ranging from 21 to 33 mm (mm). Also, the crude ethanolic extract recorded the highest antimicrobial potential against all tested microorganisms except *Bacillus cereus* when compared with the extract fractions and antimicrobial standards (Fluconazole and gentamycin). The GC–MS/MS analysed of different *Bidens* fractions revealed the presence of various phyto-components. Totally 26 constituents were identified in *Bidens aurea* from all the four analysed fractions. Ethyl acetate: ethanol (60:40) fraction has a recorded the highest number of (10) phyto-constituents, while Ethyl acetate: ethanol (20:80) fraction has lower number of (3) phytoconstituents. Based on these findings, the ethanolic extract and its fractions of *Biden aurea* exhibit promising antioxidant, antiradical, and antimicrobial activities, suggesting their potential for further investigation in biomedical application.

## Introduction

Genus *Bidens* comprises 208 to 250 species and is spread in subtropical, tropical, warm-temperate, North America, and South America [1]. Lizarazu [2] reported that Bidens L contains 280 types widespread in torrid zones and mild climate areas, and *Bidens aurea* is the most widespread species.

*Bidens aurea* (Asteraceae family) is an annual herb that produces yellow or whitish flower heads that are widely spread in the Mediterranean region [3]. Heads of *Bidens aurea* have disc and ray florets. Ibrahim [4] found *Bidens aurea* (Aiton) Sherff in Yemen, which has nine different qualitative morphological leaves that are also the same as the new *Bidens* taxon and *Bidens* in the flora of Yemen taxa. Superficial axial *Bidens aurea* leaf flower petals are light green, engraved, pubescent, and have fimbriae on the engraved margin [4].

*Bidens aurea* is mainly spread in wet regions, for example, stream banks and marshes [5]. In Egypt, Asteraceae contributes about nearly 333 taxa (10.3%) of the flora of Egypt [6]. *Bidens aurea* widespread in Asia and the Americas and has a circular chloroplast genome containing 83,909 and 18,407 base pairs in large single copy and small single copy, respectively [7].

The most phenolic components in *Bidens aurea* are catechin and quercetin; rutin, gentisic, and catechins have antimicrobial effects [8, 9]. Sharma et al. [10] stated that the phytochemical compounds are known to possess a wide range of pharmacological potential. The Asteraceae family has anticancer activity because it contains high polyphenol compounds like flavonoids, alkaloids, tannins, and steroids [11, 12].

*Bidens aurea* has been used for years in folk medicine in the treatment of gastro-duodenal diseases, gastric cytoprotective, sedative activity, rubefacients, and anti-inflammatory [13]. Bidens aurea leaves are used as a tea substitute in Spain, which has decreased inflammation and relieved digestive problems [14]. Calero [15] showed that flavonoid extract of *Bidens aurea* has a healing effect on chronic gastric ulceration in rats, which is stimulated by using 5% acetic acid when using a low concentration of diethyl ether extract (62.5 mg/kg body) after 14 days.

*Bidens aurea* has flavonoid examples such as *aurones* and chalcones [16]. *Bidens aurea* has an antiulcergenic effect against different trial effects such as stress from cold and necrotizing factors [3, 16] de la Lastra [3] demonstrated that *Bidens aurea* flowers have gastro protective effects by activating mucus glycoprotein and lowering mucosal microvascular permeability concentration through the complex prostaglandin-dependent mechanism in 250 mg/kg of flavonoid extraction pre-treatment in 120 min.

The aim of the present research is to study the chemical constituents of the extract and semi-purified fractions from the *Bidens aurea* plant and investigate the biological activities of crude extracts and their fractions as an antimicrobial as well as their effectiveness as an antioxidant effect.

## Materials and methods

### Chemicals, reagents and instruments

Pure solvents (hexane, ethyl acetate, ethanol and methanol) were obtained from E. Merck Co. (Darmstadt, Germany). Gallic acid and ascorbic acid (Vitamin C), 2, 2 diphenyl-1-picrylhydrazyl (DPPH), 2, 2'- azino-bis (ethylbenzthiazoline-6-sulfonic acid (ABTS^.+^) were obtained from Sigma-Aldrich (St. Louis, MO, USA). Spectroscopic measurements were conducted using UV–Vis spectrophotometer (Spectronic Helios Gamma, Thermo Scientific).

### Collection of plant materials

Whole plant samples of *Bidens aurea* (family: Asteraceae) were collected from the nursery of the faculty of Pharmacy, Cairo University, Giza, Egypt, during spring. The official identification was carried out by Prof. Dr. Rim Hamdy, a member of Cairo University's Herbarium and Professor of Taxonomy and Flora in the Department of Botany and Microbiology, Faculty of Science, Cairo University. The specimen was identified as *Bidens aurea* (Aiton) Sherff (Family: *Asteraceae*), using floral keys to compare the species gathered for the study with verified specimens housed in Cairo University Herbarium (CAI) under the number CAI 104.531.02.

### Extraction and samples preparation

The collected plant sample was cleaned and left to dry in shade for one week, then the dried sample was powdered and stored in brown bottle The dried powdered plant sample (100 g dry weight) was extracted three times with 500 ml of cold water (distilled water at room temperature for 12 h), hot water (distilled water at 90 °C for 15 min) and ethanol 70% for 12 h with shaking then filtered. The percentage of extraction yield was evaluated. Each extract was used for antioxidant effect assessment by DPPH and ABTS methods and total phenolic content. The most promising extract (70% ethanolic extract) was fractionated by column chromatographic analysis.

### Total phenolic content

Total phenolic content (TPC) of three extracts was conducted using the Folin-Ciocalteu method as described by Singleton and Rossi [17]. Gallic acid was used as a standard phenolic compound. The extracts were prepared in triplicate at a concentration of 1000 ppm. 0.1 ml of extract was added to 0.75 mL of tenfold diluted Folin-Ciocalteu reagent and mixed well. After 5 min, standing at room temperature, 0.75 mL of sodium carbonate (6% w/v) was added to each tube then mixed and incubated for 90 min. The absorbance at 725 nm by UV/Vis spectrophotometer was determined. A gallic acid (100–500 ppm) standard curve was plotted. Total phenolic contents of each extract were determined as mg gallic acid equivalent per g dry weight (GAE/g D. W.). Using the following formula:

$$\mathrm C={\mathrm C}_{\mathrm G}\;\mathrm V\;{\mathrm D}_{\mathrm f\;}/\mathrm m\;\mathrm{where},\;\mathrm C:\;\mathrm{total}\;\mathrm{phenolic}\;\mathrm{content}\;(\mathrm{mg}\;\mathrm{GAE}/\mathrm{gD}.\mathrm W),\;\mathrm{CG}:\;\mathrm{calibration}\;\mathrm{curve}\;\mathrm{Gallic}\;\mathrm{acid}\;\mathrm{concentration}\;(\mathrm\mu g/\mathrm{ml}),\;\mathrm V:\;\mathrm{volume}\;\mathrm{of}\;\mathrm{the}\;\mathrm{extract}\;(\mathrm{ml}),\;{\mathrm D}_{\mathrm f\;}:\;\mathrm{dilution}\;\mathrm{factor},\;\mathrm{and}\;\mathrm m:\;\mathrm{weight}\;\mathrm{of}\;\mathrm{the}\;\mathrm{dry}\;\mathrm{plant}\;\mathrm{sample}\;(\mathrm g).$$

### Antioxidant effect

#### DPPH radical scavenging activity

Antioxidant ability was conducted according to Blois [18] using 1, 1-diphenyl, 2-picrylhydrazyl free radical (DPPH^•^). Various concentrations of each sample (200, 400, 600, 800 and 1000 ppm in 100 µl) were added to 900 µl of DPPH solution in concentration of 0.1 mmol in methanol. After incubation at room temperature for 30 min, the absorbance was determined at 517 nm. Vitamin C was used as standard antioxidant material. The inhibition present was calculated according to the following equation:

$$\%\;\mathrm{inhibition}\;=\;\left[\left(\mathrm{control}\;\mathrm{absorbance}-\;\mathrm{tested}\;\mathrm{sample}\;\mathrm{absorbance}\right)/\mathrm{control}\;\mathrm{absorbance}\right]\times100$$

#### ABTS radical scavenging activity

The radical antioxidant activity of each extract against ABTS^·+^ radical was determined using the method of Re [19]. The ABTS solution was prepared by adding 7 mmol/L of ABTS solution to 2.45 mmol/L of potassium persulphate; the mixture was kept in the dark for 16 h at room temperature. ABTS prepared solution was diluted with ethanol to an absorbance of 0.70 ± 0.02 at 734 nm. Each sample (200 µl) in concentrations of 200, 400, 600, 800 and 1000 ppm was mixed with 2 ml of ABTS solution. After incubation at room temperature for 6 min, the absorbance at 734 nm was recorded. The ABTS scavenging ability was determined by the following equation:

$$\;\%\;\;\mathrm{inhibition}\;=\;\left[\left(\mathrm{control}\;\mathrm{absorbance}\;-\;\mathrm{tested}\;\mathrm{sample}\;\mathrm{absorbance}\right)/\mathrm{control}\;\mathrm{absorbance}\right]\times100\rbrack\ \mathrm{The}\;\mathrm{concentrations}\;(\mathrm{ppm})\;\mathrm{required}\;\mathrm{to}\;\mathrm{scavenge}\;50\;\%\;\mathrm{of}\;\mathrm{DPPH}\;\;\mathrm{and}\;\mathrm{ABTS}^+\mathrm{radicals}\;({\mathrm{IC}}_{50})\;\mathrm{were}\;\mathrm{estimated}.\;$$

### Colum chromatographic analysis

The fractionation of the ethanolic extract was carried out by the sequential purification through column chromatography. Column, 40 cm length, 2.5 cm diameter, was packed with 150 g column chromatography 60–120 mesh silica gel. Five grams of dried 70% ethanolic extract of *Bidens aurea* was ground with silica gel and placed on the packed column. This column was gradually eluted with different solvent mixtures starting with 100% hexane then polarity increased by in sequence with ethyl acetate and ethanol in the ratio of 80:20, 60:40, 40:60, 20:80 and 0:100 (hexane: ethyl acetate then ethyl acetate: ethanol). The fractions were collected separately then the solvent was removed for further analysis.

### Antimicrobial activity

The antimicrobial susceptibility tests were performed on ethanolic extract (70%) and its promising fractions (ethyl acetate (100%), ethyl acetate: ethanol (20:80), ethyl acetate: ethanol (60:40) and Ethanol (100%)) according to NCCLS recommendations [20]. Screening tests regarding the inhibition zone were conducted by the well diffusion method [21] against six strains of microorganisms; *Candida albicans* (ATCC 10221) as a fungal strain *Staphylococcus aureus* (ATCC 6538) and *Bacillus cereus* (ATCC 33018) as gram-positive bacteria and *Salmonella typhi* (ATCC 6539), *Escherichia coli* (ATCC 8739), and *Klebsiella Pneumonia* (ATCC13883) as gram- negative bacteria. Fluconazole and gentamycin were used as positive controls for fungi and bacteria, respectively at a concentration of 10 mg/ml and dimethyl sulfoxide (DMSO) used as a negative control.

The agar plates (Mueller–Hinton agar plates for bacteria and malt agar plates for fungi) were inoculated by spreading the microbial inoculum from colonies grown overnight over the entire agar surface. The hole with a diameter of 6 mm is punched aseptically with a sterile cork borer, and a volume (100µL) of the antimicrobial drug and different extracts dissolved in DMSO at concentration of (50 mg/ml) was introduced into the well. During incubation, the antimicrobial agents exhibit growth inhibition of the tested microbial strains. The inhibition zone was measured around each well after 24 h at 37 °C in mm beyond well diameter (6 mm).

### Identification of phytoconstituents using GC–MS/MS analysis

The GC–MS/MS analysis of the 70% ethanolic extract promising fractions (Ethyl acetate (100%), Ethyl acetate: ethanol (60:40), Ethyl acetate: ethanol (20:80) and Ethanol (100%)) were performed in order to identify the phytochemical composition, using GC–MS/MS. Column content 5%—phenyl methyl polysiloxane (HP-5 MS, Agilent) and column (capillary, 30 m × 0.25 mm i. d. and 0.25 μm film thicknesses. Helium was the carrier gas with flow rate of 1 ml/min. The oven temperature was programmed from 55 °C for 3 min and next pending programmed to 280 °C at a rate of 11 °C/min. The injection temperature was 220 °C, and the detector temperature was (220 °C). The injection volume was 1 μl in the mode of splitless. The Mass operating parameters were 70 eV and 280 °C for ionization potential and interface temperature, respectively.

The selected ion monitoring in Scan mode was applied using m/z at the start and end mass of 40 to 600. The component identification was conducted according to computer matching of their mass spectra compared with those of the authentic compounds by NIST and WILEY library and the fragmentation pattern of the mass spectral data with those revealed by the literature reviews [22].

### Statistical analysis

Values were presented as means ± SEM. Statistical analysis was performed using the “costat” computer statistic program. The one-way ANOVA is used as a statistical analysis to compare the values followed by student-Newman Keuls test. The value of probability less than 5% (P < 0.05) was considered statistically significant. Pearson’s correlation coefficient was conducted to find the relationship between the total phenolic contents of different extracts and antioxidant activity.

## Results and discussion

### Extract yield of *Bidens aurea*

Medicinal plants can act as biochemical factories; including bioactive phytochemicals, which exert multipotent effects. *Bidens* sp. was extracted using three different individual solvents (cold water, hot water, and 70% ethanol). The obtained results in Table 1 show that the ethanolic extract recorded the optimal solvent for extraction with a high extract yield, followed by hot water, then cold water (20.84, 18.69, and 17.21% respectively). These results may be due to the polarity and ability of ethanol for the extraction of most secondary metabolites present in plant cells, followed by hot water due to the ability of temperature for the extraction of some high-molecular-weight chemical compounds when compared with cold water extract. This result was in agreement with Yeasmen and Islam [23] who reported that ethanol is a suitable solvent for phytochemical extraction due to its ability to extract a high amount of phytochemicals from various waste types. Ethanol is a suitable solvent for phytochemical extraction because it can extract both polar and non-polar lipids, resulting in higher extraction yields.

**Table 1:** Extract yield (g/100 g) and total phenolic contents (as mg GAE/g) of different extracts from *Bidens aurea* plant

Plant extract	Extract yield (g/100 g)	Total phenolic content (mg GAE/g dried sample)
Cold water	17.21b ± 0.85	10.54c ± 0.03
Hot water	18.69 b ± 0.47	12.63b ± 0.12
Ethanolic	20.84a ± 0.41	28.89a ± 0.17

Data are represented as mean ± SE (*n* = 3). Means with different letters (a, b and c) within the same column shows significant differences (*p* < 0.05)

### Phenolic content of *Bidens aurea*

Phenolics include different types of bioactive compounds. These compounds take part in the regulation of many physiological processes in plants and are used as a potential source for improving human health as well. One of the most important characteristics of plant phenols is their antioxidant activity, which is exerted by their chemical structure. Total phenolic content (mg GAE/g dried sample) of cold water, hot water, and ethanolic (70%) extracts was evaluated by the Folin-Ciocalteu method of the *Bidens aurea* plant sample (Table 1). Results revealed that the total phenolic content was significantly high in 70% ethanolic extract of *Bidens aurea* which amounted to 28.89 ± 0.17 mg GAE g^−1^ dried sample. This result was a sign of the probable increase in bioactivity of the ethanolic extract. The results agreed with those reported by Jiménez-Moreno [24], who found that the aspect that generally had the biggest impact on the extraction of various bioactive chemicals was the extraction solvent's ethanol content. At 40 °C and 50% ethanol, the extracts exhibited the highest level of total phenolic components and antioxidant activity.

### Antioxidant activity of different crude extracts from *Bidens aurea*

DPPH and ABTS assays are widely used to evaluate the free radical scavenging activity of various antioxidant compounds and crude extracts. The DPPH and ABTS methods detect the scavenging properties of antioxidant compounds due to their hydrogen or electron donation. Nagah [25] concluded that the radical scavenging activity was enhanced in a dose-dependent manner.

The antioxidant effects of different extracts from *Bidens aurea* were evaluated using DPPH and ABTS assays. The results shown in Tables 2 and 3, indicated that the used assays were correlated and moreover revealed a dose-dependent effect.

**Table 2:** Antioxidant activity (%) of different extracts from *Bidens aurea* and ascorbic acid as a natural antioxidant standard against DPPH radical at different concentrations

**Conc. (µg/ml)**	**Plant extract**			**AO standard** **vitamin C**	**LSD (0.05)**
	**Cold water extract**	**Hot water extract**	**Ethanolic extract**		
200	2.87 d ± 0.18	6.23 c ± 0.32	22.96b ± 0.18	88.16a ± 0.25	0.78
400	7.46 d ± 0.06	15.68c ± 0.15	29.05b ± 0.40	93.28a ± 0.17	0.76
600	12.77 d ± 0.28	16.92c ± 0.38	41.31b ± 0.58	94.79a ± 0.26	1.29
800	13.23 d ± 0.06	20.81c ± 0.60	53.48b ± 0.44	94.65a ± 0.13	1.24
1000	14.75 d ± 0.22	30.92c ± 0.51	61.71b ± 0.22	95.12a ± 0.29	1.08
**Mean**	**10.22 d ± 0.06**	**18.11c ± 0.37**	**41.70b ± 0.25**	**93.20a ± 0.14**	0.77
*IC_**50**_	**3013.00a ± 38.11**	**1686.82b ± 34.53**	**756.13c ± 5.61**	**29.25 d ± 0.23**	84.35

Data are represented as mean ± SE (*n* = 3). Means with different letters (a, b and c) within the same row shows significant differences (*p* < 0.05)

**Table 3:** Antioxidant activity (%) of different extracts from *Bidens aurea* and ascorbic acid as a natural antioxidant standard against ABTS radical at different concentrations

**Conc. (µg/ml)**	**Plant extract**			**AO standard** **vitamin C**	**LSD (0.05)**
	**Cold water extract**	**Hot water extract**	**Ethanolic extract**		
200	5.14c ± 0.62	3.76 d ± 0.24	14.09b ± 0.22	97.82a ± 0.08	1.15
400	10.15 d ± 0.27	26.91c ± 0.41	48.34b ± 0.43	97.55a ± 0.21	1.12
600	17.65 d ± 0.80	35.91c ± 0.31	65.06b ± 0.47	99.36a ± 0.09	1.6
800	18.03 d ± 0.36	38.77c ± 0.23	73.61b ± 0.44	99.18a ± 0.21	1.05
1000	23.27 d ± 0.32	43.55c ± 0.34	87.29b ± 0.56	99.32a ± 0.08	1.2
**Mean**	**14.85 d ± 0.04**	**29.78c ± 0.02**	**57.68b ± 0.33**	**98.65a ± 0.04**	0.55
*IC_**50**_	**2065.64a ± 49.54**	**1021.97b ± 9.26**	**530.46c ± 3.58**	**24.41 d ± 0.10**	82.38

Data are represented as mean ± SE (*n* = 3). Means with different letters (a, b and c) within the same row shows significant differences (*p* < 0.05)

The obtained results revealed that the antioxidant activity of different *Bidens* extracts was higher against the ABTS radical method when compared with the obtained results with DPPH radical methods. Even so, the results of these methods (DPPH and ABTS) were found to be strongly correlated with correlation coefficient of 0.99. The correlation between antioxidant activity IC_50_ and total phenolic content (mg GAE g-1 dried sample) was in a strong negative correlation with correlation coefficient of −0.87 by DPPH method and −0.81 ABTS method. These results proved the phenolic compounds contribution in the antioxidant effects of different used extracts. Moreover, the ascorbic acid as a natural antioxidant standard gave the highest antioxidant activity against both radical assays at different concentrations (200–1000 µg/ml) when compared with all plant extracts.

Also, these results revealed that the *Bidens aurea* ethanolic extract exhibited the highest antioxidant ability against both radicals (DPPH and ABTS) with 61.71 ± 0.22 and 87.29 ± 0.56% at 1000 µg/ml followed by hot water extract 30.92 ± 0.51 and 43.55 ± 0.34% respectively as shown in Tables 2 and 3 in comparison with ascorbic acid which recorded 95.12 ± 0.29 and 99.32 ± 0.08% at 1000 µg/ml against DPPH and ABTS respectively.

The IC_50_ of different extracts was inversely proportional to the antioxidant activity of crude plant extracts and antioxidant standard. The data in Tables 2 and 3 recorded that ascorbic acid have the lowest IC_50_ followed by ethanolic extract of *Bidens aurea* by 24.41 ± 0.10 and 530.46 ± 3.58 µg/ml respectively, against ABTS radical assay.

These findings were in accordance with Rico [9] who recorded that the *B. aurea* showed a higher antioxidant effect than BHA and BHT as synthetic antioxidant agents. In another previous research, the antioxidant effect against DPPH and ABTS of various extracts from *B. pilosa* from different locations has been investigated in which the leaves methanolic extract of *B. pilosa* showed IC_50_ values of 80.45 and 171.6 µg/mL, respectively [26].

### Antioxidant activity of different column fractions from the ethanolic extract of *Bidens aurea*

The ethanolic extract of *Bidens aurea* showed 6 column fractions (starting from ethyl acetate 100% to ethanol 100%). The antioxidant activities of these fractions were determined using two antioxidant methods (DPPH and ABTS). The obtained results in Tables 4 and 5 revealed that the same trend was observed for all tested fractions against both DPPH and ABTS. The fraction no. 6 (ethanol 100%) recorded the highest antioxidant activity by 90.40 ± 0.13 and 94.34 ± 0.18% at 1000 µg/ml respectively followed by fraction no. 5 (ethyl acetate: ethanol 20:80) by 82.61 ± 0.19 and 87.00 ± 0.48% at 1000 µg/ml respectively.

**Table 4:** Antioxidant activities (%) of semi-purified fractions of ethanolic extract from *Bidens aurea* against DPPH at different concentrations

Conc. (µg/ml)	Column fractions						LSD (0.05)
	EA	EA:ET (80:20)	EA:ET (60:40)	EA:ET (40:60)	EA:ET (20:80)	ET	
200	8.04e ± 0.14	9.72 d ± 0.18	17.76b ± 0.3	6.34f ± 0.07	15.56c ± 0.46	31.99a ± 0.70	1.05
400	13.45e ± 0.20	17.88 d ± 0.45	22.13c ± 0.28	7.16f ± 0.40	31.78b ± 0.24	50.46a ± 0.35	1.06
600	41.24c ± 0.43	27.63e ± 0.07	37.73 d ± 0.61	9.49f ± 0.38	51.41b ± 0.30	77.61a ± 0.22	1.10
800	54.46c ± 0.33	34.58e ± 0.10	52.34 d ± 0.50	13.24f ± 0.17	69.85b ± 0.27	84.42a ± 0.08	0.91
1000	67.81c ± 0.06	41.92 d ± 0.49	68.43c ± 0.22	16.00e ± 0.23	82.61b ± 0.19	90.40a ± 0.13	1.06
Mean	37.00 d ± 0.12	26.35e ± 0.16	39.68c ± 0.11	10.45f ± 0.07	50.24b ± 0.04	66.98a ± 0.09	0.37

Data are represented as mean ± SE (*n* = 3). Means with different letters (a, b and c) within the same row shows significant differences (*p* < 0.05)

**Table 5:** Antioxidant activities (%) of semi-purified fractions of ethanolic extract from *Bidens aurea* against ABTS at different concentrations

Conc. (µg/ml)	Column fractions						LSD (0.05)
	EA	EA:ET (80:20)	EA:ET (60:40)	EA:ET (40:60)	EA:ET (20:80)	ET	
200	28.48b ± 0.71	6.79e ± 0.48	8.03 de ± 0.96	9.06 d ± 0.80	22.93c ± 0.92	35.22a ± 0.13	1.63
400	58.45a ± 0.33	6.61 d ± 0.63	16.18c ± 0.28	15.23c ± 0.71	54.33b ± 0.16	53.06b ± 0.84	1.27
600	75.71b ± 0.80	9.38e ± 0.28	25.91 d ± 0.59	25.03 d ± 0.73	72.58c ± 0.05	78.52a ± 0.44	1.22
800	84.46b ± 0.28	16.85e ± 0.16	33.35 d ± 0.52	32.80 d ± 0.73	77.87c ± 0.30	92.17a ± 0.13	1.06
1000	87.79b ± 0.11	19.66e ± 0.58	45.53c ± 0.56	38.35 d ± 0.43	87.00b ± 0.48	94.34a ± 0.18	1.04
Mean	66.98b ± 0.21	11.86f ± 0.19	25.80 d ± 0.24	24.09e ± 0.46	62.94c ± 0.14	70.66a ± 0.13	0.60

Data are represented as mean ± SE (*n* = 3). Means with different letters (a, b and c) within the same row shows significant differences (*p* < 0.05)

**Fig. 1:**
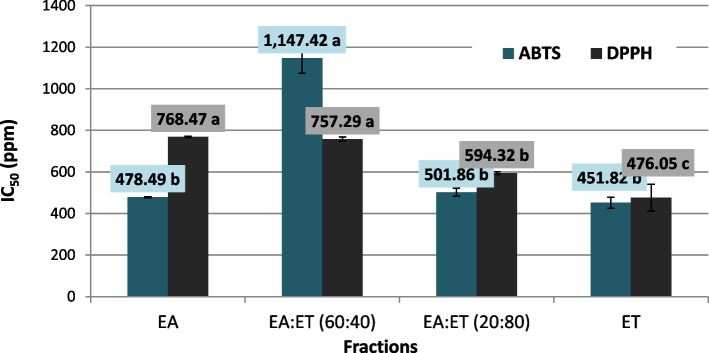
IC_50_ of the most fractions of 70% ethanolic extract of *Bidens aurea* plant sample. IC_50_ = concentration required to inhibit 50% of DPPH and ABTS activities

The IC_50_ for these fractions were arranged from the lowest to the highest as the follows: ET, EA: ET (20:80), EA, EA:ET (60:40), EA:ET (40:60), and EA:ET (80:20) as shown in Fig. 1. From the obtained data we can conclude that the antioxidant activity of ethanolic fractions increased with the increasing the polarity of the used solvent as the mobile phase and these results may be due to the presence of polar antioxidant compounds such as phenolic compounds, pigments and low molecular weight alkaloids which are extracted with the most polar solvents.

Also, these data may be due to the active ingredients present in each fraction, as mentioned in the GC–MS/MS results (Table 7). The obtained results revealed that the ethyl acetate: ethanol (60:40) fraction contains some phenolic derivatives such as flavone, 5-hydroxy-3, 3’, 4’, 6, 7-pentamethoxy, and 7, 3’, 4’, 5’-tetrahydroxyflavone. Moreover, the ethyl acetate: ethanol (20:80) fraction contains 6-ethoxy-4-methylcoumarin and vitexin. However, the ethanol (100%) fraction contains 3, 4-dihydroxymandelic acid, β-carotene, propyl gallate, and vitexin; all of these compounds recorded high antioxidant activity.

These findings were in accordance with the result found by Pillai [27] who indicated a moderate to strong radical scavenging effects and ferric reducing power beside the presence of significant amount of phenolic and flavonoid compounds of *B. pilosa* various extracts, in which the leaf and stem- bark methanolic extracts possessed the highest effect on DPPH.

### Antimicrobial activity of ethanolic extract and their column fractions of Bidens aurea

The growth inhibition zone (IZ) diameters were shown in Table 6. The obtained results revealed that the ethanolic extract of *Bidens aurea was* extract were effective against all tested microorganisms (1 fungi and 5 bacteria) with an IZ diameters ranging from 21 to 33 mm (mm). Also, the given data observed that the crude ethanolic extract recorded the highest antimicrobial potential against all tested microorganisms except *Bacillus cereus* when compared with the extract fractions and antimicrobial standards (Fluconazole and gentamycin).

**Table 6:** Antimicrobial activity (as mm in diameter) of ethanolic extract (70%) and its promising fractions of *Bidens aurea*

Pathogenic microorganism		Samples						LSD (0.05)
		**Ethanolic crude extract**	**Column fractions**				**Positive** **control**	
			**EA**	**EA: ET** **(20:80)**	**EA: ET** **(60:40)**	**ET**		
**Fungi**	***Candida albicans***	22a ± 0.58	19.67bcd ± 0.33	19 cd ± 0.58	20.33abc ± 0.33	21.33ab ± 0.33	17.67 d ± 0.88	1.68
**Gram positive bacteria**	***Staphylococcus aureus***	33.33a ± 0.88	31.33ab ± 0.33	29.67b ± 1.20	29.33b ± 0.67	28.66b ± 0.88	29b ± 0.58	2.48
	***Bacillus cereus***	21.33b ± 1.20	19.33b ± 0.67	27.67a ± 0.33	26.33a ± 1.20	25.33a ± 0.88	25.33a ± 0.67	2.72
**Gram negative bacteria**	***Salmonella typhi***	30a ± 0.58	28a ± 0.00	25b ± 0.58	21.33c ± 0.33	20.33c ± 0.67	20c ± 1.15	2.01
	***Escherichia coli***	29.33a ± 0.33	22.67b ± 0.88	23.67b ± 0.33	21.66b ± 0.33	22.33b ± 0.67	23.33b ± 0.88	1.92
	***Klebsiella Pneumonia***	23a ± 1.00	23a ± 1.15	24.33a ± 0.33	25a ± 0.58	26.67a ± 0.33	24.67a ± 0.88	2.41

Data are represented as mean ± SE (*n* = 3). Means with different letters (a, b and c) within the same row shows significant differences (*p* < 0.05). Gentamycin is used for bacteria and fluconazole is used for fungi as a positive control

**Fig. 2:**
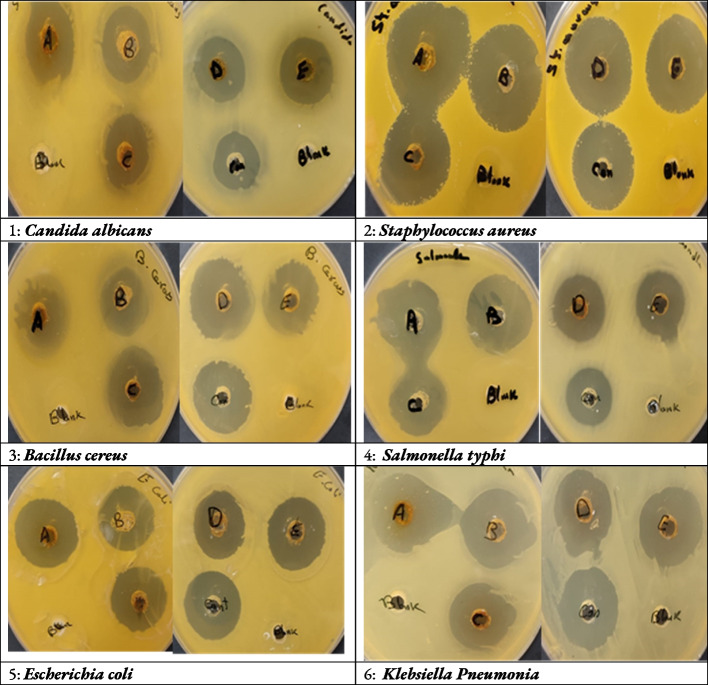
Microorganisms growth inhibition caused by crude ethanolic extract (**A**), its 100% ethyl acetate fraction (**B**), (20:80) ethyl acetate: ethanol fraction (**C**), (60:40) ethyl acetate: ethanol fraction (**D**), 100% ethanol fraction (**E**) and fluconazole for fungi or gentamycin for bacteria as a positive control (con) of some food poisoning bacterial strains (*Candida albicans*, *Staphylococcus aureus*, *Bacillus cereus*, *Salmonella typhi*, *Escherichia coli* and *Klebsiella Pneumonia*

The crude ethanolic extract gave the highest activity against *Staphylococcus aureus* by IZ diameter 33.33 ± 0.88 followed by the ethyl acetate fraction and EA: ET (20:80) and EA: ET (60:40) by 31.33 ± 0.33, 29.67 ± 1.20 and 29.33 ± 0.67 mm (Table 6 and Fig. 2).

In general, the crude extract was more active against the tested microorganisms and recorded the higher when compared with antimicrobial standards. These results may be due to the synergistic effect between the active ingredients present in this extract. The antimicrobial effect of plant extracts is tested by adding the extracts to an agar medium and monitoring their spread, there are many factors that affect the IZ diameters. The effect of extracts on the agar environment may be to activate or inhibit the growth of microbes or have no effect [28].

Also, these data may be due to the active ingredients present in each fraction as mentioned in GC–MS/MS results (Table 7). The obtained results revealed that the Ethyl acetate (100%) fraction contains 1-Tricosanol; however, the Ethanol (100%) fraction contains Propyl gallate and Salsoline compounds which recorded the antimicrobial properties. Phenolic acids like propyl gallate can be transported through the cell membrane of bacteria and disturb it through decreased pH of the cytoplasm and denaturation of protein [29]. Vitexin has an antimicrobial effect on *P. aeruginosa* by inhibiting biofilm formation, and it has a cytotoxicity effect on macrophages on the host [30].

**Table 7:** Phyto-chemical components (Relative %) in various column fractions of *Bidens aurea* utilizing GC-MS/MS

SN	R.t.	Compound name	Chemical structure	Relative %	Biological activities	References
**Ethyl acetate (100%) fraction**						
1	13.08	3-(1,1-Dimethylallyl)scopoletin		11.73	NA	
2	13.33	4-Acetylbenzoic acid		68.53	NA	
3	20.05	1-Tricosanol		6.10	antibacterial activity anticancer activity	[25, 31]
4	20.74	1-Heptacosanol		6.35	NA	
5	21.23	Elaidic acid		7.29	NA	
**Ethyl acetate: ethanol (60:40) fraction**						
1	13.08	Isocalamenediol		4.73	NA	
2	13.32	p-Cresol, 2-tert-butyl-		35.13	NA	
3	13.49	Flavone, 5-hydroxy-3,3',4',6,7-pentamethoxy-		12.17	anti-allergic activity antioxidant activity anticancer activity	[32, 33]
4	13.59	7,3',4',5'-Tetrahydroxyflavone		4.66	antioxidant activity	[34]
5	14.37	Isophytol		6.28	antimicrobial activity	[35]
6	14.56	Erucic acid		11.65	anti-inflammatory activity	[36]
7	14.64	5β,7βH,10α-Eudesm-11-en-1α-ol		5.90	antidiabetic activity	[37]
8	14.71	Octadecanoic acid		7.77	NA	
9	16.19	p-Menthane-1,2-diol		7.32	NA	
10	22.4	Benzoic acid, 2,6-dihydroxy (γ-resorcylic acid)		4.39	NA	
**Ethyl acetate: ethanol (20:80) fraction**						
1	13.33	6-Ethoxy-4-methylcoumarin		84.93	antioxidant activity	[38]
2	20.05	Vitexin		7.24	*antioxidant activity*-*antinflammation activity*- anticancer activity	[39–42]
3	21.72	(S)-(-)-Citronellic acid		7.82	NA	
**Ethanol (100%) fraction**						
1	13.47	3,4-Dihydroxymandelic acid		20.41	Radical scavenging activity	[43]
2	14.73	β Carotene		3.06	Radical scavenging activity-anticancer	[43–46]
3	15.19	Ledol		4.00	NA	
4	15.71	6,7,8-Trimethoxycoumarin		5.52	*gastroprotective activity*	[47]
5	16.29	Salsoline		31.01	inhibition of a-amylase and antifungal activity	[47, 48]
6	17.58	Hexa-hydro-farnesol		24.99	NA	
7	21.45	Propyl gallate		7.99	*antioxidant activity*-*antimicrobial activity*- anticancer activity	[49–54]
8	23.23	Vitexin		3.02	*antioxidant activity*-*antinflammation activity*- anticancer activity	[39–42]

### GC–MS/MS analysis of *Bidens aurea* fractions

The GC–MS/MS analysis of different *Bidens* fractions revealed the presence of different phyto-components. The phyto-components of all fractions are shown separately in Table 7. Totally 26 constituents were identified in *Bidens* sp from all the four analysed fractions. Ethyl acetate: ethanol (60:40) fraction has recorded the highest number of (10) phytoconstituents, while in Ethyl acetate: ethanol (20:80) fraction fewer than 3 phyto-constituents were shown, which contain high and low components.

The major constituents were 4-acetylbenzoic acid (68.53%) and 3-(1, 1-dimethylallyl) scopoletin (11.73%) in ethyl acetate (100%) fraction. Moreover, The ethyl acetate: ethanol (60:40) fraction showed the presence of p-cresol, 2-tert-butyl- (35.13%) and Erucic acid (11.65%). But in ethyl acetate: ethanol (20:80), the fraction showed presence of 6-ethoxy-4-methylcoumarin was shown to be 84.93%. However, ethanol (100%) fraction has recorded 3, 4-dihydroxymandelic acid by 20.41%.

Our results were agreed with Abdulkader [55] who identified components of the ethanolic extract of *Bidens pilosa* by GC–MS/MS and found the extract contains highly effective compounds likes (tannins, flavonoids, saponins, alkaloids, and sterols. These compounds have bioactive and medicinal effects, so leaves of *Bidens pilosa* have the same benefit.

## Conclusion

From the obtained results, we can conclude that the crude aqueous and alcoholic extracts of the Bidens aurea plant have different bioactive compounds, especially phenolic compounds. The biological activity of these crude extracts and semi-purified fractions exhibited high antioxidant and anti-radical effects at different concentrations. Also, the obtained data recorded the high antimicrobial activity of crude plant extracts against all tested microorganisms except *Bacillus cereus* when compared with the extract fractions and antimicrobial standards (fluconazole and gentamicin). The obtained results also revealed that the ethyl acetate: ethanol (60:40) fraction contains some phenolic derivatives, such as flavone, 5-hydroxy-3,3',4',6,7-pentamethoxy, and 7,3',4',5'-tetrahydroxyflavone. Based on these findings, it is concluded that *Biden aurea* ethanolic extract and its fractions can be widely used as potential antioxidant, antiradical and antimicrobial agents for biomedical applications. Further study will be conducted for separation and identification of active ingredients from the most promising semi-purified fraction according to the obtained data, in addition to identifying the mode of action of these separated compounds using the molecular docking technique.

## Data Availability

The specimen was identified as *Bidens aurea* (Aiton) Sherff (Family: Asteraceae), using floral keys ‎to compare the species gathered for the study with verified specimens housed in Cairo University Herbarium (CAI) under the number CAI 104.531.02.

## Abbreviations

DPPH: 2,2-Diphenyl- 1-picrylhydrazyl
ABTS: 2,2-Azinobis (3-ethylbenzothiazoline- 6-sulfonic acid)
GC–MS/MS: Gas chromatography/mass spectrometry
TPC: Total phenolic content
